# P-1588. Impact of the COVID-19 Pandemic on Sepsis due to Serratia: Trends in Incidence, Mortality, and Hospital Resource Utilization — A National Inpatient Sample Analysis (2016–2022)

**DOI:** 10.1093/ofid/ofaf695.1767

**Published:** 2026-01-11

**Authors:** Mohamed Barghout, Michelle Lee, Nouf K Almaghlouth

**Affiliations:** Lifespan, Warren Alpert Medical School of Brown University, Providence, Rhode Island; Brown University Health Rhode Island Hospital, Providence, Rhode Island; University of Pittsburgh Medical Center , Pittsburgh, PA

## Abstract

**Background:**

Serratia species are emerging causes of healthcare-associated sepsis, with growing concerns about antimicrobial resistance and high mortality. The influence of the coronavirus disease 2019 (COVID-19) pandemic on national trends of incidence, outcomes, and healthcare utilization in sepsis due to Serratia remains unclear.

**Table 1:**
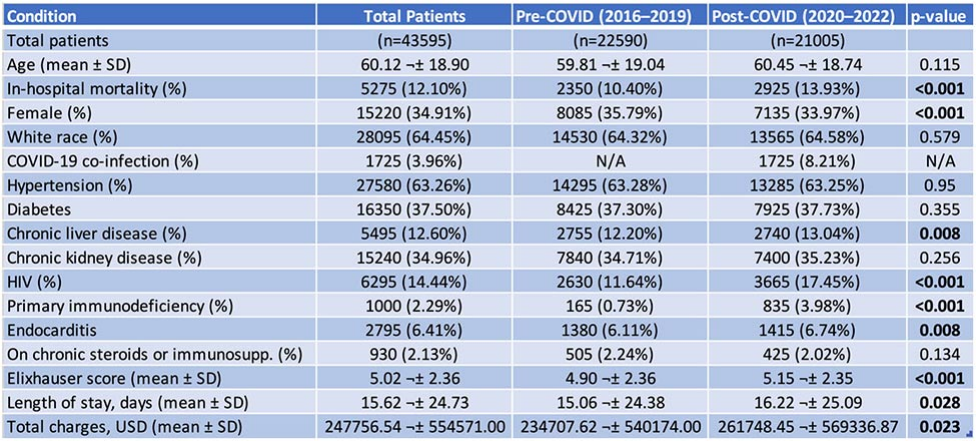
Baseline characteristics and outcomes of patients before and during the COVID-19 era (2016–2022)

**Figure 1:**
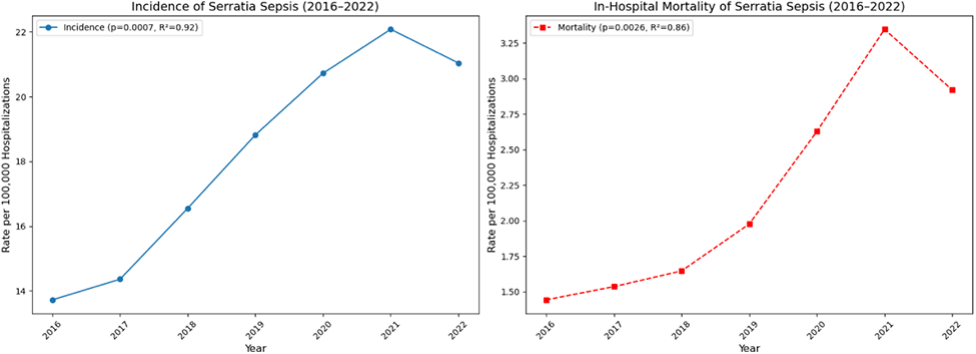
Temporal trends in the incidence and in-hospital mortality of sepsis due to Serratia (2016–2022)

**Methods:**

We analyzed the National Inpatient Sample (NIS) from 2016 to 2022 to identify adult hospitalizations with sepsis due to Serratia and relevant comorbidities using ICD-10 codes. The study period was stratified into pre-COVID (2016–2019) and post-COVID (2020–2022) groups. Weighted national estimates were used to assess annual incidence and In-hospital mortality per 100,000 hospitalizations. Hospital charges were adjusted for inflation using the Medical Care Consumer Price Index. Subgroup analysis compared outcomes between COVID-19 co-infected and non-co-infected patients during the post-COVID period. Temporal trends were analyzed using linear regression. Categorical variables were compared using Chi-square or Fisher's exact test, and continuous variables using t-tests or analysis of variance.

**Results:**

Among 43,595 sepsis due to Serratia hospitalizations, incidence rose from 13.7/100,000 (2016) to 21.0/100,000 (2022; peak 22.1 in 2021, p=0.0007). Mortality increased from 1.44/100,000 pre-COVID to 2.92/100,000 post-COVID (peak 3.34, p< 0.001). Post-COVID era had longer length of stay (LOS) (16.2 vs. 15.1 days, p=0.028) and higher charges ($261,748 vs. $234,708, p=0.023). COVID co-infection (8.2%) was linked to higher mortality (36.8% vs. 11.9%, p< 0.001), LOS (29.8 vs. 15.0 days, p< 0.001), and charges ($521,009 vs. $238,640, p< 0.001).

**Conclusion:**

The incidence and mortality of sepsis due to Serratia have significantly increased in the United States during the COVID-19 pandemic, accompanied by greater healthcare resource utilization. These findings highlight the critical need for enhanced infection control and focused strategies to mitigate the burden of sepsis due to Serratia in the post-pandemic era.

**Disclosures:**

All Authors: No reported disclosures

